# Role of primary tumor volume and metastatic lymph node volume in response to curative effect of definitive radiotherapy for locally advanced head and neck cancer

**DOI:** 10.1186/s40001-024-01691-0

**Published:** 2024-02-04

**Authors:** Weiling Mao, Tao Zhang, Longhao Li, Siyun Peng, Huiying Gong, Minmin Li

**Affiliations:** 1 Department of Oncology, The First Affiliated Hospital of Chongqing Medical University, No. 1 Youyi Road, Yuzhong District, Chongqing, 400016 China; 2 Department of Oncology, Chongqing University Three Gorges Hospital, Chongqing, China; 3 Department of Sociology, Indiana University, Bloomington, IN USA

**Keywords:** Head and neck squamous cell carcinoma, Primary tumor volume, Lymph node volume, Definitive radiotherapy, Tumor response

## Abstract

**Background:**

Studies have shown mixed results concerning the role of primary tumor volume (TV) and metastatic lymph node (NV) volume in response to the curative effect of definitive radiotherapy for locally advanced head and neck squamous cell carcinoma (LAHNSCC).

**Objective:**

We aimed to evaluate the impact of TV and NV on the efficacy of radical radiotherapy in LAHNSCC patients, with the goal of guiding individualized therapy.

**Patients and methods:**

Patients with LAHNSCC who received radical radiation therapy and were reexamined within 6 months post-therapy from January 2012 to December 2021 were selected. The volumes of the primary tumors and metastatic lymph nodes were calculated by software and then were divided into a large TV group *vs* small TV group and a large NV group *vs* small NV group according to the relationship with the median. Additionally, patients who received concurrent chemoradiotherapy (CCRT) or not were divided into the CCRT group and the radiotherapy (RT) group. Patients with lymph node metastasis were divided into node concurrent chemotherapy (N-CCRT) group and a node metastatic chemotherapy (N-RT) group according to whether they received concurrent chemotherapy or not. The volume shrinkage rate (VSR), objective response rate (ORR), local control rate (LCR) and overall survival (OS) were recorded and analyzed.

**Results:**

96 patients were included in the primary tumor volume group, and 73 patients were included in the metastatic lymph node group. Receiver operating characteristic (ROC) curves were constructed for objective remission (OR) endpoints, and a volume threshold was defined for TV and NV patients. The threshold primary tumor volume was 32.45 cm^3^, and the threshold metastatic lymph node volume was 6.05 cm^3^.The primary TV shrinkage rates of the small TV and the large TV groups were basically the same, *P* = 0.801. Similarly, the ORR and LCR were not significantly different between the small TV group and the large TV group (*P*_ORR_ = 0.118, *P*_LCR_ = 0.315). Additionally, the TV shrinkage rate did not significantly differ between the CCRT group and the RT group, *P* = 0.133. Additionally, there was no significant difference in ORR or LCR in CCRT group (*P*_ORR_ = 0.057, *P*_LCR_ = 0.088). However, the metastatic lymph node volume shrinkage rate in the small NV group was significantly greater than that in the large NV group (*P* = 0.001). The ORR and LCR of the small NV subgroup were significantly greater than those of the large NV subgroup (*P*_ORR_ = 0.002, *P*_LCR_ = 0.037). Moreover, compared with that of the N-RT group, the NV shrinkage rate of the N-CCRT group was 84.10 ± s3.11%, and the shrinkage rate was 70.76 ± s5.77% (*P* = 0.047*).* For the ORR and LCR, the N-CCRT group and N-RT group were significantly different (*P*_ORR_ = 0.030, *P*_LCR_ = 0.037). The median OS of the whole group was 26 months. However, neither TV/NV nor concurrent chemotherapy seemed to influence OS.

**Conclusion:**

Primary tumor volume is not a prognostic factor for the response to curative effect radiotherapy in LAHNSCC patients. Nevertheless, metastatic lymph nodes are a prognostic factor for the response to curative effect radiotherapy in LAHNSCC patients. Patients with smaller lymph nodes have better local control.

## Introduction

Head and neck squamous cell carcinoma (HNSCC) is the sixth most common cancer worldwide [1, 2]. Most patients are diagnosed at a locally advanced stage. Despite aggressive treatment involving surgery, radiation, and chemotherapy, the recurrence rates are still approximately 50% in locally advanced HNSCC [3].

As we all know, tumor and node stages determined by tumor size, lymph nodes spread and presence of metastasis (refer to TNM staging) could be a powerful predictor of curative effect [4] for patients who receive radiotherapy. Primary tumor volume and metastatic lymph node volume have not been well studied in predicting the efficacy of HNSCC patients. However, with the improvement of imaging technology, volume theoretically provides a more accurate description of solid tumors. The biological basis for this is a relationship between the malignant tumor cell number and tumor control, as the relationship is nearly linear [5, 6]. Previous studies suggested that both primary tumor volume and metastatic lymph nodal volume were both have a part predictive effect but there is no consensus [7–9]. Primary tumor volume may be a powerful predictor of recurrence and overall survival [10]. Shang-Wen Chen et al. reported that the patient's primary gross tumor volume (PGTV) is a strong outcome predictor for hypopharyngeal cancer with treatment of concurrent chemoradiotherapy (CCRT) [11]. Robert et al. found that the primary tumor volume determined by CT was significantly correlated with the local recurrence rate in tonsil cancer patients [12]. However, Janssens et al. found that in patients with stage T2–4 laryngeal cancer receiving radiotherapy, no correlation was detected between the primary tumor volume and local control, regional control and metastasis free survival [13].

In the meanwhile, some researchers have assessed the impact of lymph nodal volume on local control and overall survival, which supposed to be a prognostic factor independent of American Joint Cancer Committee (AJCC) N staging [9, 14, 15]. Studies found that the metastatic lymph node volume was a significant predicator in univariate analysis of local control [14, 15]. Robert et al. study suggested that the metastatic lymph node volume determined by CT was significantly correlated with the regional prognosis [13]. Verger et al. found that a total lymph node volume > 14 cm^3^ would lead to a high risk of regional recurrence rate [16], while Jakobsen et al. study found that total lymph node volume > 100 cm^3^ was a cut-off value to determine the 2 year DFS [17]. However, an Indian study showing there was no correlation between metastatic lymph node volume and response to RT [18].

Based on these heterogeneous results, we planned to explore the role of primary tumor volume and metastatic lymph node volume in response to curative effect of definitive radiotherapy for LAHNSCC and tried to guide individualized therapy for different groups of people.

## Methods

### Patient selection and clinical data collection

A retrospective review was conducted of all patients with HNSCC who visited the First Affiliated Hospital of Chongqing Medical University from January 2012 to December 2021. The inclusion criteria for patients were as follows: had a tumor site in the oral cavity, oropharynx, hypopharynx or larynx; an AJCC stage of locally advanced; or who received radical radiotherapy. The patients who were included in our study had imaging data (computed tomography (CT), CT/magnetic resonance, MR) available for review. The exclusion criteria for patients were as follows: did not undergo imaging examination after radiotherapy, could not be evaluated, or had a follow-up time longer than half a year after the first radiotherapy. The data we collected included patient sex, tumor node metastasis classification (TNM) stage, disease status, etc. Each patient underwent radiation therapy at a total dose of 70 Gy; the treatment was completed in 33 or 35 sessions, with each dose of 2.12 Gy or 2 Gy, lasting for 6.5–7 weeks. Cisplatin or carboplatin was included.

### Grouping

Receiver operating characteristic (ROC) curves for objective remission (OR) endpoints and define a volume threshold corresponding to TV and NV. According to the threshold tumor volume, we divided patients into small primary tumor volume group, and large primary tumor volume group (small TV group and large TV group for short); Similarly, patients were divided into small metastatic lymph node group and large metastatic lymph node group (small NV group and large NV group for short) according to the size of the threshold metastatic lymph node. Patients were divided into a CCRT group and an RT group according to whether concurrent chemotherapy was added to the radiotherapy. Patients with lymph node metastasis were divided into node concurrent chemotherapy (N-CCRT) group and a node-radiotherapy (N-RT) group according to whether they received CCRT or not.

### Calculation of primary tumor volume and metastatic lymph node volume

We selected two radiologists who were unaware of the patient’s results to perform structural imaging (CT or MR). The eclipse treatment planning system (TPS) (Eclipse 15.6, VARIAN, USA) software was used to outline the primary tumor and metastatic lymph nodes, and the software automatically generated the outline volume. Calculating the volume with the built-in calculation system. The volume shrinkage rate of primary tumor is defined as (volume of primary tumor before treatment—volume of primary tumor after treatment)/volume of primary tumor before treatment * 100%. Similarly, the volume shrinkage rate of metastatic lymph nodes = (volume of metastatic lymph nodes before treatment—volume of metastatic lymph nodes after treatment)/volume of metastatic lymph nodes before treatment * 100%.

According to RECIST 1.1 standard, we divide the disease status into complete remission, partial remission, stable disease and progress disease. We define local control (LC) as complete remission, partial remission or stable disease on CT or MR within half a year after radiotherapy. Complete remission + partial remission on CT or MR within half a year after radiotherapy is defined as objective remission. Local control rate (LCR) refers to the proportion of patients under local control in the total number of patients. Objective remission rate (ORR) refers to the proportion of patients with objective remission in the total number. Overall survival (OS) is defined as the time interval between the date of diagnosis and the date of death or the date of the last follow-up, in months.

### Statistics

Statistics were performed using IBM SPSS Statistics 26.0 (IBM, Armonk, NY). Data were entered by 3 researchers and confirmed by cross-checking. The counting data were analyzed by Chi-square test. *Z*-test, binomial test, binary logistic regression and linear regression were used for comparison between multiple groups and two groups. First, the Kolmogorov–Smirnov method is used to test the normal distribution of the measurement data. The measurement data close to the normal distribution are expressed as mean ± standard deviation, and the analysis of variance is used for comparison among groups; The measured data of non-normal distribution are expressed by median (quartile) and interdigit interval [M (Q1, Q3)]. Kruskal–Wallis H test was used for comparison between multiple groups and between two groups. The difference was considered statistically significant when *P* < 0.05. K–M function was used to calculate survival analysis.

## Results

### General information

We initially screened 96 patients in total, the patient’s gender, tumor stage, disease status, OS and other data characteristics are shown in Table 1.

**Table 1:** Patient data characteristics

Patient data characteristics	*n*		
		Group	
		Small-T	Large-T
Total number of patients	96	68	28
Sex (%)			
Female	17.7%(17)	12	5
Male	82.3%(79)	56	23
Tumor staging			
III	47	47	0
IV	49	21	28
Concurrent chemoradiotherapy	45	34	11
Radiotherapy	51	34	17
Disease state (RECIST1.1)			
Complete remission	7.3%(7)	6	1
Partial remission	70.8%(68)	50	18
Stable disease	6.3%(6)	3	3
Disease progression	15.6%(15)	9	6
Threshold volume of primary tumor (cm^3^)	32.45		
Threshold volume of metastatic lymph node (cm^3^)	6.05		
T-grouping situation			
	96	68	28
Volume range of primary tumo r(cm^3^) [min,max]	[2.1,174.4]		
N-grouping situation			
	73	32	41
Volume range of lymph node (cm^3^) [min,max]	[0.5,97.9]		
OS (month) (median, [IQR])	26 [19,41]		
VSR (%) (median, [IQR])			
VSR-T	56.47 [33.09,77.61]		
VSR-N	89.56[65.87,100]		

*IQR* inter-quartile range, *VSR* volume shrinkage rate, *OS* overall survival, *T* tumor, *N* metastatic lymph nodes

### The shrinkage rate, ORR and LCR focusing on the primary tumor volume

According to thethreshold, we divided patients into small TV group and large TV group. The median TV shrinkage rate of the small TV group was consistent with the large TV group, *P* = 0.801(Table 2). Additionally, the ORR of the small TV group and the large TV group were similar, *P* = 0.118(Table 2), the LCR of the small TV group was 72.8% *v* 78.6% of the large TV group, *P* = 0.315.

**Table 2:** Volume retraction rates, ORR, and LCR in the primary tumor volume group

Item	Volume shrinkage rate (%)	*P*	ORR	*P*	LCR	*P*
T-group	*T*-test	0.801	Chi-square test	0.118	Chi-square test	0.315
Small-T	51.66		82.4% (56/68)		72.8% (59/68)	
Large-T	49.44		67.9% (19/28)		78.6% (22/28)	
CCRT or not	*T*-test	0.133	Chi-square test	0.057	Chi-square test	0.088
CCRT	57.38		86.7% (39/45)		91.1% (41/45)	
RT	45.40		70.6% (36/51)		78.4% (40/51)	

The tumor volume shrinkage rates of two groups were basically the same, *P* = 0.133 (Table 2) and the ORR were not statistically different in CCRT group and RT group *P* = 0.057. Also, the LCR of the CCRT group and RT group were basically same, *P* = 0.088 (Table 2)*.*

We tried to find out whether CCRT had an impact on the result trend of primary tumor volume, so we used logistic regression analysis to analyze the T grouping and whether CCRT and RT or not. When the outcome index is ORR, *P* = 0.168 for TV group, and *P* = 0.080 for CCRT, both of which have no statistical difference. Similarly, when the outcome index is LCR, *P* = 0.402 for TV group and *P* = 0.113 for CCRT, there is no statistical difference between the two groups.

### The shrinkage rate, ORR and LCR focusing on the lymph node volume

The median metastatic lymph NV shrinkage rate of the small NV group was obviously higher than that of the large NV group. There was a significant difference, *P* = 0.001 (Table 3). As well, the ORR of the small NV group was significantly higher than that of the large NV group, *P* = 0.002 (Table 3). The LCR of the small NV group was obviously higher than that of the large NV group, *P* = 0.037 (Table 3).

**Table 3:** Volume retraction rate, ORR, and LCR in the metastatic lymph node volume group

Item	Volume shrinkage rate (%)	*P*	ORR	*P*	LCR	*P*
N-group	*T*-test	0.001*	Chi-square test	0.002*	Chi-square test	0.037*
Small-N	88.81		90.6% (29/32)		90.6% (29/32)	
Large-N	68.71		58.5% (24/41)		70.7% (29/41)	
CCRT or not	*T*-test	0.47*	Chi-square test	0.030*	Chi-square test	0.037*
N-CCRT	84.10% ± s3.11%		83.8% (31/37)		89.2% (33/37)	
N-RT	70.76% ± s5.77%		61.1% (22/36)		69.4% (25/36)	

PS. *indicates statistical significance

In NV group, the average volume shrinkage rate of metastatic lymph nodes in N-CCRT group is significant higher than that in N-RT group (P = 0.047) (Table 3). Similarly, ORR of N-CCRT group and N-RT group *P* = 0.030 (Table 3). LCR was 89.2% in N-CCRT group and 69.4% in N-RT group, *P* = 0.037 (Table 3).

Similarly, we used logistic regression analysis to analyze the situation of N group and whether CCRT are synchronized. We found that when ORR was used as the evaluation index, *P* = 0.006 in NV group, odds ratio (OR) was 0.148; There was significant difference between whether CCRT (*P* = 0.049), OR was 3.240. When LCR is the evaluation index, *P* = 0.060 for NV group and *P* = 0.059 for CCRT, there is no statistical difference between the two groups.

### Survival outcomes

In our study, the median OS was 26 months, the shortest OS was 8 months, and the longest OS was 104 months. Total follow-up number of people were 57. With death as the primary endpoint, K-M function survival analysis was used. The results showed that there was no significant difference in survival time between small TV group and large TV group (*P* = 0.477). There was no significant difference in survival time between small NV group and large NV group, *P* = 0.229. Similarly, there was no significant difference in survival time between CCRT group and RT group, *P* = 0.749. Using COX regression with volume and CCRT as covariates, both T and N groups showed no statistical significance.

## Discussion

Therapy includes active treatment, for example, surgical radiotherapy, chemotherapy, and targeted therapy. The curative effect of treatment for patients with HNSCC is still unsatisfactory [3, 19]. The efficacy of radical radiotherapy in LAHNSCC patients needs to be improved. Therefore, our study focused on whether the curative effect of radical radiotherapy was related to tumor volume.

We found that primary tumor volume had no effect on the shrinkage rate, ORR or LCR. Therefore, we assumed that, no matter how large the primary tumor volume was, the efficiency of the existing radical radiotherapy was equivalent and did not change with the addition of synchronous chemotherapy. This finding is similar to the findings of Mendenhall et al. [20], who suggested that the local control of patients with oropharyngeal and hypopharyngeal tumors who underwent CCRT and the LCR without serious complications were not affected by tumor volume. Moreover, we found that the addition of CCRT did not change the trend of local control in patients according to the primary tumor volume grouping. Hamauchi et al. [21] also reported that, in patients with high-risk stage II laryngeal cancer, the LCRs of the small TV and large TV groups were not significantly different, regardless of whether CCRT was added. In definitive radiotherapy for LAHNSCC, CCRT can improve survival outcomes compared to RT alone [22]. Several studies have shown that CCRT can improve the LCR in HNSCC patients. Tang et al. reported that the LCR in HNSCC patients in the CCRT group was significantly greater than that in the RT group (HR = 0.46, 95% CI = 0.37–0.57, *P* < 0.001) [23]. Mak et al. reported that CCRT based on cisplatin provides greater local control in LAHNSCC patients [24]. However, in our study, there were no significant differences in the primary volume shrinkage rate, ORR, or LCR between the CCRT and RT groups. We found that the absolute value of the primary tumor volume regression rate in the CCRT group was significantly greater than that in the RT group; however, unfortunately, *P* = 0.133 was not significantly different between the two groups. Similar results were also observed for the ORR and LCR (*P*_ORR_ = 0.057, *P*_LCR_ = 0.088). We speculate that this may be due to the insufficient sample size, which resulted in the results not reaching statistical significance.

Interestingly, for metastatic lymph nodes, the treatment response of the small NV group was better than that of the large NV group, regardless of the shrinkage rate of metastatic lymph nodes, ORR or LCR, and this trend did not change with the addition of synchronous chemotherapy. This finding is similar to that of Chiesa Estomba et al. [25], in which the size of the positive lymph nodes increased, the risk of regional control failure increased. Moreover, some studies have shown that local control of HNSCC patients improves with decreasing diameter and volume of metastatic lymph nodes [26]. Therefore, can we assume that we may need to re-examine existing radical radiotherapy strategies to improve the short-term treatment efficacy in patients with large metastatic lymph nodes? First, our study showed that the shrinkage rate, ORR and LCR of metastatic lymph nodes in the CCRT group were significantly better than those in the RT group. These findings suggest that this approach is an effective method for increasing concurrent chemotherapy efficacy. Other studies have reported that CCRT can help reduce the size of metastatic lymph nodes in HNSCC patients [27, 28]. In addition, Klausner et al. [29] suggested that lymph node dissection after CCRT in HNSCC patients would result in better local control. Dua B's opinion is that patients with N2 stage disease and patients with a metastatic lymph node volume of 15 cm^3^ have a higher risk of regional recurrence, and cervical lymph node dissection can be considered after radiotherapy and chemotherapy [30]. In the Dua B study, the radiation dose to the metastatic lymph nodes was 56 Gy (1.6 Gy/time, 35 times). Therefore, for patients with large metastatic lymph nodes, can the combined treatment mode of CCRT + neck lymph node dissection be used as a new type of processing method? In addition, since the size of metastatic lymph nodes has a significant impact on the efficacy of RT, it is particularly important to determine an appropriate threshold. In Hermans et al [12] study, patients were divided into four groups according to the quartile of metastatic lymph node volume. The same method was also used in Verger et al [16] study. In our study, patients were divided into a small N group and a large N group according to the median volume of metastatic lymph nodes (7.6 cm^3^). Hermans [12] suggested that the local control and OS of patients with a metastatic lymph node volume < 14.5 cm^3^ were better than those with a metastatic lymph node volume > 14.5 cm^3^. Dua B [30] reported that the optimal lymph node volume threshold for OS was 15 cm^3^. According to Ahmed I [31], patients with a TV < 30 cc, an NV < 4 cc, or a T + N < 50 cc had better CR. We used the cut-off value of the ROC curve with ORR as the indicator. Therefore, the best estimation threshold needs to be determined in further research.

Therefore, the current standard concurrent radiotherapy and chemotherapy strategy is still the best choice for patients with LAHNSCC with large primary tumor volumes in terms of short-term efficacy. For patients with large metastatic lymph nodes at initial diagnosis, the sincere cooperation of the head and neck tumor multidisciplinary treatment (MDT) team is recommended. We can boldly assume that the primary tumor should be given a radiation dose of 70 Gy, the metastatic lymph nodes should be given a radiation dose of only 50 Gy, synchronous chemotherapy should be carried out, and then selective neck lymph node dissection should be carried out. Unfortunately, there is no clear research indicating this point. This is a bold assumption that we can further verify in the future.

In our study, the primary tumor volume had no effect on OS, which is consistent with the findings of Kamal et al [32]. They found no correlation between primary tumor volume and survival outcomes in patients with T3 laryngeal cancer. Many studies have shown that the OS of HNSCC patients receiving radical radiotherapy is better in the small NV subgroup than in the large NV subgroup [12, 30]. However, in our study, although the absolute OS of patients with small NVs was greater than that of patients with large NVs, the difference was not statistically significant. In Hermans’s [12] study, the metastatic lymph node threshold was 14.5 cm^3^; in Dua’s [30] study, the metastatic lymph node threshold was 15 cm^3^; and in our study, the metastatic lymph node volume was 7.6 cm^3^. Therefore, the difference in OS may be related to the difference in metastatic lymph node volume. Lang et al. [33] reported that among HNSCC patients, the 5 year survival period was significantly prolonged in patients who underwent CCRT, but there was no significant difference in PFS or local disease-free survival (LDFS). In our study, although the median OS of patients in the CCRT group was greater than that of patients in the RT group (*P* = 0.072), the difference was not significant. Lang K’s study used a 5 year survival period, while our longest follow-up was 9 years. The study showed that the risk of death in HNSCC patients after 5 years is significantly ^greater34^, and a longer follow-up may offset the difference in OS between the two groups. Moreover, due to the long duration of follow-up, many patients were lost to follow-up, which is also a reason for the unsatisfactory OS results. Taking into account various factors may ultimately result in no significant difference in OS.

Nonetheless, our research has several limitations. Although we tried our best to contact patients, the failure rate of follow-up was 40.6%. This has a certain impact on our evaluation of long-term efficacy. We also did not determine whether patients had HPV infection, which may have had some impact on our experimental results. We plan to verify in the following experiments whether HPV infection has an impact on lymph node volume as a classification indicator. Most of our patients were from southwestern regions, such as Yunnan, Guizhou, Sichuan, Chongqing, and Hubei, which are relatively concentrated regions and may have led to biased results. In the future, we plan to further improve multicenter research to evaluate and replicate these findings. In addition, almost all of our patients received induction chemotherapy, which may have had some effect on the treatment, but unfortunately, we did not discuss the relevant issues this time.

## Conclusion

The primary tumor volume was not a factor influencing the efficacy of radiotherapy in LAHNSCC patients. The volume of metastatic lymph nodes is a factor influencing the efficacy of radiotherapy in LAHNSCC patients. Patients with a smaller metastatic lymph node volume had better local control, and this trend did not change with the addition of synchronous chemotherapy. In the future, additional research should be conducted on the different responses of primary tumor volume and metastatic lymph node volume to RT, and an improved standard treatment approach should be identified for patients with large metastatic lymph node volumes.

## Data Availability

Not applicable.
